# Purinergic Receptor P2Y2 Stimulation Averts Aortic Valve Interstitial Cell Calcification and Myofibroblastic Activation

**DOI:** 10.3390/biomedicines10020457

**Published:** 2022-02-16

**Authors:** Donato Moschetta, Enrico Di Maria, Vincenza Valerio, Ilaria Massaiu, Michele Bozzi, Paola Songia, Yuri D’alessandra, Veronika A. Myasoedova, Paolo Poggio

**Affiliations:** 1 Centro Cardiologico Monzino IRCCS, 20138 Milan, Italy; donato.moschetta@ccfm.it (D.M.); enrico.dimaria93@gmail.com (E.D.M.); vincenza.valerio@ccfm.it (V.V.); ilaria.massaiu@ccfm.it (I.M.); michele.bozzi@ccfm.it (M.B.); paola.songia@gmail.com (P.S.); yuri.dalessandra@ccfm.it (Y.D.); veronika.myasoedova@ccfm.it (V.A.M.); 2 Department of Pharmacological and Biomolecular Sciences, University of Milan, 20133 Milan, Italy; 3 Developmental Biology of the Immune System, Life & Medical Sciences (LIMES) Institute, University of Bonn, 53115 Bonn, Germany

**Keywords:** CAVS, VICs, fibro-calcification, P2Y2 receptor, 2ThioUTP

## Abstract

Rationale—Calcific aortic valve stenosis (CAVS) is a pathological condition of the aortic valve with a prevalence of 3% in the general population. It is characterized by massive rearrangement of the extracellular matrix, mostly due to the accumulation of fibro-calcific deposits driven by valve interstitial cells (VIC), and no pharmacological treatment is currently available. The aim of this study was to evaluate the effects of P2Y2 receptor (P2RY2) activation on fibro-calcific remodeling of CAVS. Methods—We employed human primary VICs isolated from CAVS leaflets treated with 2-thiouridine-5′-triphosphate (2ThioUTP, 10 µM), an agonist of P2RY2. The calcification was induced by inorganic phosphate (2 mM) and ascorbic acid (50 µg/mL) for 7 or 14 days, while the 2ThioUTP was administered starting from the seventh day. 2ThioUTP was chronically administered for 5 days to evaluate myofibroblastic activation. Results—P2RY2 activation, under continuous or interrupted pro-calcific stimuli, led to a significant inhibition of VIC calcification potential (*p* < 0.01). Moreover, 2ThioUTP treatment was able to significantly reduce pro-fibrotic gene expression (*p* < 0.05), as well as that of protein α-smooth muscle actin (*p* = 0.004). Conclusions—Our data suggest that P2RY2 activation should be further investigated as a pharmacological target for the prevention of CAVS progression, acting on both calcification and myofibroblastic activation.

## 1. Introduction

Calcific aortic valve stenosis (CAVS) is a slow and progressive pathological disorder of the aortic valve with a prevalence of 3% in the general population over 65 years of age [1]. To date, transthoracic echocardiography is the recommended initial test to diagnose CAVS [2,3,4]. It is characterized by impaired leaflet motion, restricted aortic valve area, and nonuniform leaflet thickening due to fibro-calcification processes [5]. Despite this high prevalence and associated mortality, no pharmacological treatment is currently available to slow or even halt CAVS progression. As a result, surgical (AVR) or percutaneous (TAVR) aortic valve replacement remain the only effective treatments for CAVS [6], leaving the underlying pathological molecular and cellular mechanisms, which actively drive the pathology, unsolved. Oxidative stress is among the first triggers of the pathology, leading to endothelial damage [7,8], thus paving the way to the development of local inflammation [9] and the consequent activation of valve interstitial cells (VICs) [8,10]. VICs are a mixed population of cells with a mesenchymal origin, mostly composed of fibroblast-like cells that can be easily activated under different conditions, such as oxidative stress, inflammation, and pro-fibrotic/calcific stimuli [11,12,13]. Activated VICs phenotypically switch to osteoblast- and myofibroblast-like cells and subsequently deposit fibro-calcific nodules [6,14,15]. This process leads to a vicious cycle in which fibro-calcific nodule accumulation causes an increase in leaflet stiffness that, in turn, boosts VIC activation [16]. Despite the huge number of studies aimed at defining the molecular effectors of CAVS progression, they are not yet fully discovered. Several proteins have been identified as key regulators of ectopic calcification [6,17,18]. Of note, the activation of purinergic receptor P2Y2 (P2RY2) has been linked to calcium resorption [19,20] and to tissue fibrosis [21]. This evidence paves the way for the investigation of P2RY2 as a pharmacological target for CAVS treatment. To date, no information is available on the effect of P2RY2 activation in human VICs isolated from CAVS patients. Thus, the aim of the present study was to investigate the effects of P2RY2 stimulation by 2-thiouridine-5′-triphosphate (2ThioUTP) on ectopic calcification and myofibroblastic activation of VICs isolated from calcified aortic valve leaflets.

## 2. Materials and Methods

### 2.1. Study Population

Thirty-three patients who underwent surgical aortic valve replacement (AVR) were enrolled in the study at Centro Cardiologico Monzino IRCCS. Patients’ characteristics are summarized in Table 1. We collected patients with the following preoperative inclusion criteria: isolated surgical aortic valve replacement, elective surgery, age over 18 years, left-ventricular ejection fraction > 30%, and normal sinus rhythm. Patients with prior cardiac surgery, rheumatic heart disease, endocarditis, active malignancy, chronic liver and kidney diseases, calcium regulation disorders (hyperparathyroidism, hyperthyroidism, and hypothyroidism), and chronic or acute inflammatory states (sepsis, autoimmune disease, and inflammatory bowel disease) were excluded from this study. Patients with aortic valve sclerosis (AVSc) underwent surgical AVR due to aortic valve insufficiency. The study was approved by the Institutional Review Board and Ethical Committee of Centro Cardiologico Monzino (IRCCS). All enrolled patients provided written informed consent to participate in this study. The study protocol conformed to the ethical guidelines of the 1975 Declaration of Helsinki.

### 2.2. Aortic Valve Interstitial Cell Isolation and Culture

The collected aortic valve leaflets were employed to isolate valve interstitial cells (VICs) by using a modified method previously described [22,23]. Briefly, each leaflet was incubated for 20 min at 37 °C in 2 mg/mL type II collagenase (Worthington Biochemical Corp., Lakewood, NJ, USA) in cell culture medium (advanced Dulbecco’s modified Eagle’s medium (Ad DMEM, Life Technologies, Carlsbad, CA, USA) containing 10% fetal bovine serum (FBS, Microtech, Naples, Italy), 1% penicillin (Life Technologies, Carlsbad, CA, USA), 1% streptomycin (Life Technologies, Carlsbad, CA, USA), and 1% l-glutamine (Life Technologies, Carlsbad, CA, USA)). After the endothelial cell removal, the leaflets were moved away in new fresh medium and mechanically disrupted using a scalpel, before incubating for at least 4 h in 2 mg/mL type II collagenase in cell culture medium at 37 °C to process the extracellular matrix proteins.

### 2.3. Calcification and Fibrosis Assays

Cells were seeded and treated every 2 days, changing medium every time, for 7 or 14 days with ascorbic acid (AA, 50 µg/mL) and inorganic phosphate (Pi, 2 mM), with or without 2ThioUTP (10 uM). At the end of the assay, after medium removal, we employed 0.6 M HCl to dissolve extracellular calcium. After 5 h of HCl treatment in gentle agitation, samples were collected and stored at +4 °C. Cells on the bottom of the plate were incubated a +4 °C in 0.1 M NaOH with 0.1% sodium dodecyl sulfate (SDS) for total protein quantification using bicinchoninic acid (BCA) Protein Assay (Thermo Fisher Scientific, Waltham, MA, USA). To quantify extracellular calcium, we used a calcium colorimetric assay kit (BioVision, Waltham, MA, USA), following the manufacturer’s protocol. The absorbance was detected using the Infinite^®^ 200pro (TECAN) spectrophotometer. Each datum was normalized on the total protein quantification.

For the fibrosis assays, cells were seeded and treated with 2ThioUTP (10 µM) every day without changing media for 5 days. At the end of the assay, total mRNA and protein were extracted and preserved at −20 °C until analyzed.

### 2.4. Western Blot

Western blot analysis was carried out to assess protein expression using specific antibodies against α-smooth muscle actin (αSMA; Abcam, Cambridge, UK) and α-tubulin (Novus Biologicals, Minneapolis, MN, USA) as an endogenous control. Briefly, we collected and lysed untreated or 2ThioUTP-treated cells in RIPA buffer with a protease and phosphatase inhibitor cocktail (Thermo Scientific, Waltham, MA, USA). Total protein concentration was assessed through the use of a BCA kit (Thermo Scientific, Waltham, MA, USA). Afterward, proteins separation was performed using Bolt™4–12% Bis-Tris Plus Gels (Thermo Scientific, Waltham, MA, USA); after the run, we transferred proteins onto a nitrocellulose membrane and blocked them with 5% skim milk (Sigma-Aldrich, St. Louis, MO, USA). To detect target proteins, we incubated membrane overnight at 4 °C with specific primary antibodies. Then, the membranes were washed with TBS plus 0.1% Tween-20 and incubated with IRDye-conjugated secondary antibody (LI-COR Biosciences) for 20 min. Finally, the membranes were washed to remove any excess secondary antibody and acquired using an Odyssey Infrared Imaging System (LI-COR Biosciences). Densitometric analysis was performed using the software ImageJ (Version 1.48v—National Institute of Health).

### 2.5. Reverse Transcription and Real-Time PCR

RNA extraction was performed from VICs using the Total RNA Purification Plus Kit (Norgen Biotek Corp., Thorold, ON, Canada), according to the manufacturer’s instructions. Briefly, cells were lysed in RL buffer, and gDNA was removed by a “gDNA removal column”; then, RNA was purified by an RNA extraction column, washed with ethanol 100%, and diluted in “elution solution A”. Next, 10 µg of total RNA underwent DNase treatment using a TURBO DNA-free™ Kit (ThermoFisher scientific, Waltham, MA, USA), following the manufacturer’s instructions. RNA obtained was then precipitated in pure ethanol and ammonium acetate (125 mM), washed with ethanol 70%, and resuspended in deionized water, to ensure purity before being quantified by Nanodrop One. Total RNA (1 μg) was converted into cDNA, using Luna RT supermix (New England Biolabs, Ipswich, MA, USA). Real-time PCR (qPCR) was carried out on an AB Prism 7900 HT (Applied Biosystems), according to the manufacturer’s instructions; analysis was performed using software SDS2.4 (Life Technologies, Carlsbad, CA, USA).

### 2.6. Imaging Flow Cytometry Analyses

VIC characterization was performed by an ImageStream X flow cytometer, which combines flow cytometry with microscopy technology (ImageStream X Mark II, Amnis). VICs were detached through TripLE Select (Gibco, Carlsbad, CA, USA), an enzymatic method useful to preserve the extracellular epitopes of proteins on the cell surface. Then, we fixed cells in paraformaldehyde, incubated in NH_4_Cl 50 mM to break down cell auto-fluorescence, permeabilized in saponin 0.2%, and resuspended in 100 μL of PBS with 5 mM ethylenediaminetetraacetic acid (EDTA), 1% bovine serum albumin (BSA), and 0.2% saponin. Then, cell samples were incubated for 30 min at room temperature using specific conjugated-antibodies against CD90–phicoeritrin (Becton Dickinson PharmingenTM, Franklin Lakes, NJ, USA), vimentin–AlexaFluor 488 (Becton Dickinson PharmingenTM, Franklin Lakes, NJ, USA), and DRAQ5 (eBioscience, St. Clara, CA, USA), a nuclear stain. For the P2RY2 staining, cells were detached and incubated in the same way, then stained with anti-P2RY2 (Santa Cruz Biotechnology) for 30 min, and conjugated with an anti-rabbit–AlexaFluor 647 for 30 min. Samples were then washed with FACS buffer (PBS containing 5 mM EDTA and 1% BSA) by centrifugation for 5 min at 600× *g*. Finally, cells were resuspended in FACS buffer and analyzed. A total of 30,000 events were acquired in the single cells and focus gated area. Image analysis was performed using the IDEAS 6.2 software.

### 2.7. Capillary Western Blot

The capillary Western blot was performed using WES for Simple Western (Protein Simple, BioTechne). Briefly, a total of 2 μg of protein was diluted in fluorescent master mix 5×, sample buffer 10×, and DTT, following the manufacturer’s instructions. Then, samples were thermically reduced at 95 °C for 5 min and loaded in a separation module 12–230 kDa (Protein Simple, Minneapolis, MN, USA). The running protocol was the standard one. To detect P2RY2 expression, we used anti-P2RY2 (Santa Cruz Biotechnology, Dallas, TX, USA) and anti-rabbit HRP-conjugated antibody (Protein Simple, Minneapolis, MN, USA). Image analysis was performed using Compass for Simple Western software.

### 2.8. Statistical Analyses

All continuous variables are represented as the mean ± standard error of mean (SEM), while categorical are represented as percentages (%). Statistical significance was evaluated with GraphPad Prism (version 9—San Diego, CA, USA). Variables were analyzed by Student’s *t*-test or one-way ANOVA. A *p-*value < 0.05 was deemed statistically significant.

## 3. Results

### 3.1. Valve Interstitial Cell Characterization

**Figure 1:**
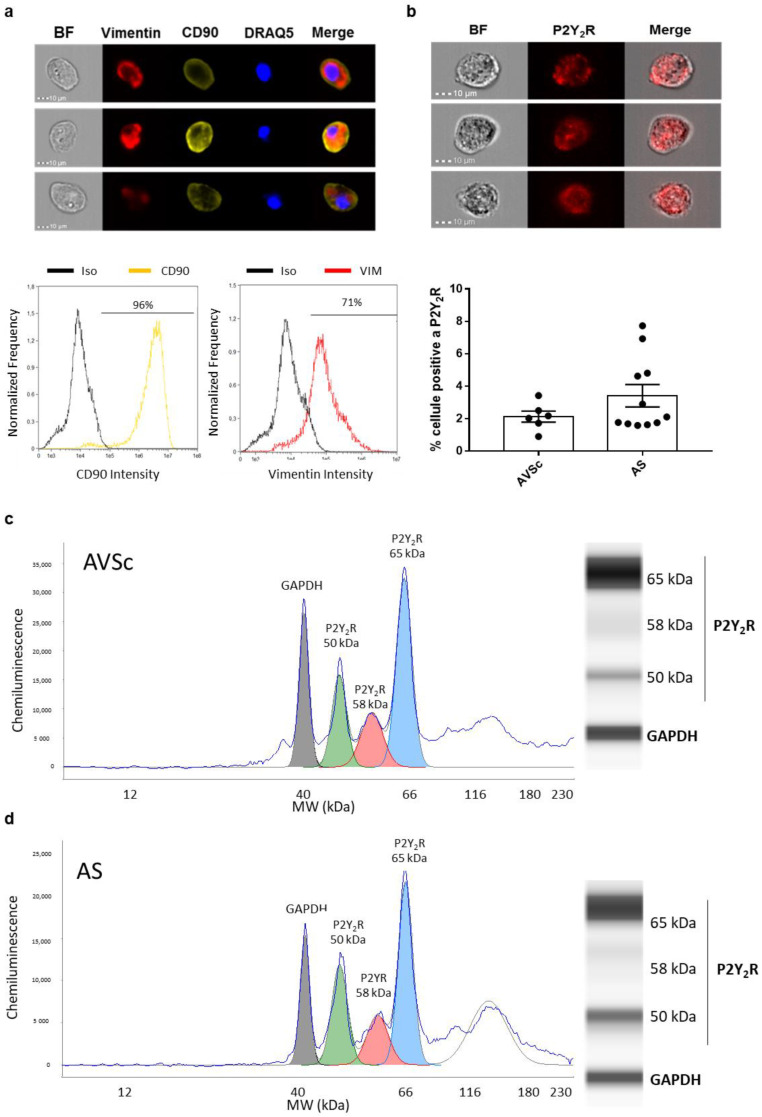
Valve interstitial cellc characterization. (**a**) Representative images of vimentin and CD90 expression of VICs (nucleus stained with DRAQ5) with relative quantifications (red and yellow line, respectively; black line: isotype control; *n* = 4). (**b**) Representative images showing VICs stained for P2RY2 (red) and relative percentage of positive cells in sclerotic (AVSc; *n* = 6) and stenotic (AS; *n* = 11) patients. (**c**,**d**) Representative quantification strategy by Compass for SW v3.1.7 (Protein Simple) and illustrative image of capillary Western blot showing P2Y2 receptor (P2RY2) and GAPDH expression of AVSc (*n* = 6) and AS (*n* = 6) patients. Scale bar: 10 µm. BF: bright field.

The isolated VICs expressed CD90 and vimentin at an intracellular level (Figure 1a). As expected, almost all nucleated cells (stained with DRAQ5) expressed CD90, indicating their mesenchymal origin, and a total of 71% of VICs co-expressed vimentin. We verified the P2RY2 expression in isolated VICs in terms of both mRNA, by qPCR (mean Ct: 32.6 ± 0.8; *n* = 15), and protein, analyzed by capillary Western blot (WES). WES evaluation revealed three different bands at 50, 58, and 65 kDa, probably due to post-translational modifications of P2RY2 [24]. After normalization on GAPDH, the 65 kDa band resulted the most expressed one (ANOVA *p* < 0.0001; *n* = 12; Figure 1c,d), without differences between AVSc and AS patients (2.97 ± 1.34 vs. 3.08 ± 1.21, respectively). The other two bands were not different between AVSc and AS patients (50 kDa 1.08 ± 0.36 vs. 1.15 ± 0.50, respectively, and 58 kDa 1.01 ± 0.37 vs. 0.86 ± 0.63, respectively). However, only a low percentage of cells expressed P2RY2 at the membrane level, as shown by imaging flow cytometry analysis performed on AVSc (2.1% ± 0.8%; *n* = 6) and AS patients (3.4% ± 2.3%; *n* = 11), with no statistical differences between the two groups (*p* = 0.21; Figure 1b). Thus, we tested the effects of 2ThioUTP, a potent and selective agonist of P2RY2, on calcium deposition and myofibroblastic activation of human VICs isolated from calcified aortic valve leaflets.

### 3.2. Extracellular Calcium Potential of VICs Treated with 2ThioUTP

**Figure 2:**
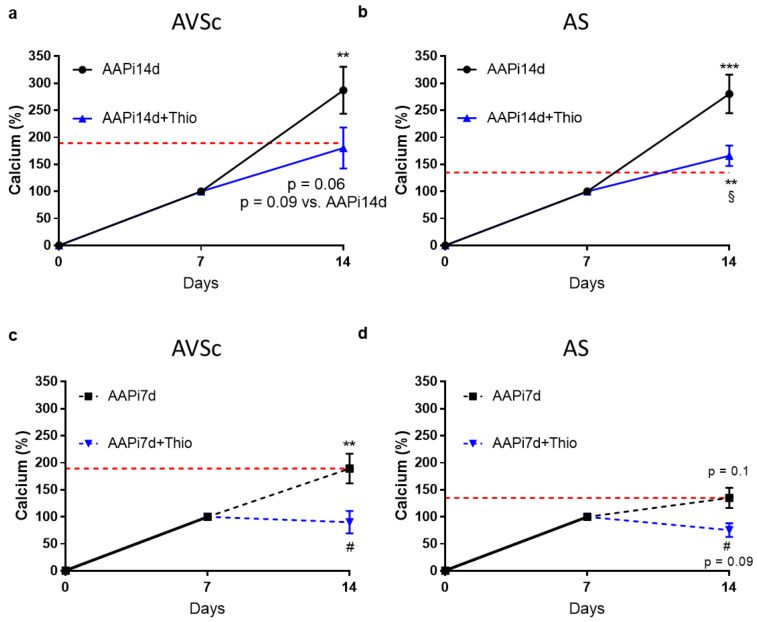
P2RY2 activation reduces extracellular calcification of human valve interstitial cells. Extracellular calcium quantification under pro-calcific condition for 14 days (AAPi14d), with the addition of 2ThioUTP 10 µM (AAPi14d + Thio) in (**a**) sclerotic (AVSc; *n* = 6) and (**b**) stenotic (AS; *n* = 5) patients. Extracellular calcium quantification under pro-calcific condition for only the first 7 days (AAPi7d), with the addition of 2ThioUTP 10 µM (AAPi7d + Thio) in (**c**) sclerotic (AVSc; *n* = 6) and (**d**) stenotic (AS; *n* = 5) patients. Data were calculated by setting the calcium amount at day 7 in pro-calcific conditions to 100% (*n* = 11). The red line is set to the mean extracellular calcification of AVSc (**a**–**c**) and AS (**b**–**d**) VICs on the 14th day in pro-calcific conditions for the first 7 days (AAPi7d). Black denotes the inner control; blue denotes treatment with 2thioUTP. Solid line denotes the pro-calcific medium; dashed line denotes normal medium. ** *p* < 0.01, *** *p* < 0.001 vs. calcification on day 7; # *p* < 0.05 vs. AAPi7d; $ *p* < 0.05 vs. AAPi14d; if not specified, *p*-values reported are vs. calcification on day 7.

To verify the extent of extracellular calcification, we employed an in vitro assay with ascorbic acid (AA; 50 µg/mL) and inorganic phosphate (Pi; 2 mM), widely recognized pro-calcific stimuli. The calcification was induced for 7 days, shown in Figure 2 as 100%, or 14 days, while 2ThioUTP (10 µM) was administered on the seventh day until the end of the experiment (14 days, *n* = 6 AVSc and 5 AS). Under continuous pro-calcific stimulus, the activation of P2RY2 led to a mild reduction in VIC extracellular calcification in AVSc patients (AAPi14d + 2ThioUTP: 180% ± 93% vs. AAPi14d: 287% ± 107%; *p* = 0.09; Figure 2a) and to a significant inhibition of VIC calcification potential in AS patients (AAPi14d + 2ThioUTP: 166% ± 43% vs. AAPi14d: 280% ± 80%; *p* = 0.005; Figure 2b). Similarly, when pro-calcific stimulus was interrupted on the seventh day, P2RY2 activation drastically reduced the extracellular calcification compared to its own control both in AVSc (AAPi7d + 2ThioUTP: 90% ± 51% vs. AAPi7d: 189% ± 67%; *p* = 0.02; Figure 2c) and AS patients (AAPi7d + 2ThioUTP: 75% ± 28% vs. AAPi7d: 135% ± 42%; *p* = 0.03; Figure 2d). Of note, the calcification under 2ThioUTP treatment, in pro-calcific medium for 14 days, was comparable to the condition in which calcification was induced only for the first 7 days without 2ThioUTP administration in AVSc (AAPi14d + 2ThioUTP: 180% ± 93% vs. AAPi7d: 189% ± 67%; *p* = 0.85) and AS patients (AAPi14d + 2ThioUTP: 166% ± 43% vs. AAPi7d: 135% ± 42%; *p* = 0.28).

Taken together, these data suggested that the activation of P2RY2 by 2ThioUTP prevents the extracellular calcium deposition in pathological VICs even under continuous stressed conditions.

### 3.3. ThioUTP Effects on Myofibroblastic Activation of VICs

**Figure 3:**
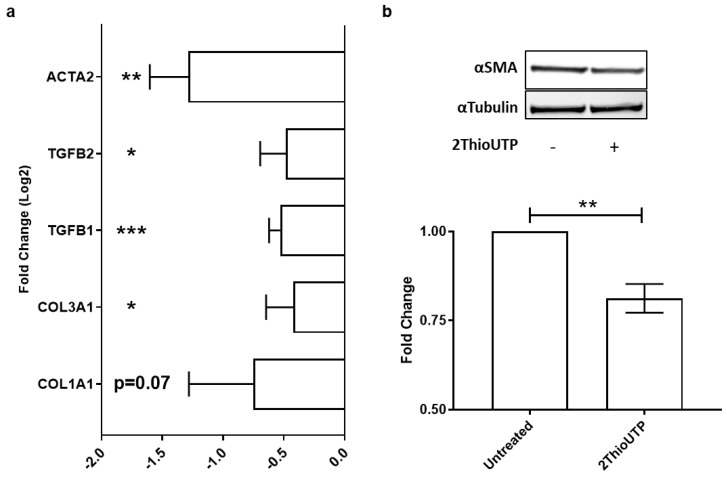
P2RY2 activation reduces human valve interstitial cell fibrosis. (**a**) Relative expression of collagen 1A1 and 3A1 (COL1A1 and 3A1), transforming growth factor β1 and 2 (TGFB1 and 2), and actin α2 (ACTA2) genes in VICs treated with 2ThioUTP 10 µM for 5 days (*n* = 3). (**b**) α-Smooth muscle actin (αSMA) protein expression and relative quantification (*n* = 4). Data are expressed as logarithm base 2 of the fold change (log_2_(FC)) or fold change (FC) vs. untreated cells and are shown as the mean ± standard error. * *p* < 0.05, ** *p* < 0.01, *** *p* < 0.001 vs. untreated.

In order to evaluate the impact of P2RY2 stimulation on myofibroblastic activation, an important and underestimated feature of CAVS, we treated VICs with 2ThioUTP (10 µM) for 5 days in normal medium. We evaluated the expression of several pro-fibrotic genes by qPCR, and we found that 2ThioUTP treatment was able to significantly reduce their expression (*n* = 3; Figure 3a). In particular, we found that collagen 1A1 (COL1A1) was not significantly reduced by treatment, but we can appreciate a negative trend after the treatment with 2ThioUTP (−0.75 ± 0.5 log_2_(FC) vs. untreated; *p* = 0.07). All other screened genes were downregulated by 2ThioUTP: collagen 3A1 (COL3A1 −0.42 ± 0.2 log_2_(FC); *p* = 0.03), transforming growth factor β (TGFβ1 −0.53 ± 0.1 log_2_(FC); *p* = 0.0006), TGFβ2 (−0.48 ± 0.2 log_2_(FC); *p* = 0.02), and actin α2 (ACTA2 −1.28 ± 0.3 log_2_(FC); *p* = 0.002). Since the upregulation of ACTA2 mRNA and, thus, αSMA is a widely recognized marker of VIC activation (myofibroblast-like phenotype), we evaluated the protein expression. We found that αSMA expression was significantly downregulated by 2ThioUTP treatment (0.81 ± 0.04 FC vs. untreated; *p* = 0.004; *n* = 4; Figure 3b).

All these data corroborate the hypothesis that P2RY2 stimulation inhibits myofibroblastic gene and protein expression in AS patients.

## 4. Discussion

Aortic valve replacement is one of the most common procedures performed in cardiac surgery, and there is no available pharmacological approach for CAVS treatment able to revert the accumulation of ectopic calcific nodules. For this reason, it is imperative to investigate new pharmacological targets capable of blocking CAVS progression and possibly reverting the pathological phenotype. The activation of P2RY2 by 2ThioUTP has been reported to prevent extracellular calcification, and its administration has already been tested in a mouse model, proving its safety (e.g., no effects on bone mineral density, inflammation, and lipid profile) [19]. The same authors showed that the depletion of P2RY2 resulted in an ineffectiveness of 2ThioUTP on ectopic calcification of mouse and healthy human VICs [19]. Our data showed, for the first time to our knowledge, that the stimulation of P2RY2 by 2ThioUTP attenuates the extracellular calcification and myofibroblastic activation—an important and underestimated pathological feature of CAVS—of VICs isolated from human calcified aortic valve leaflets. Thus, P2RY2 stimulation could be implemented at the earliest stage of CAVS (i.e., aortic valve sclerosis; AVSc), which is an essential step toward the end-stage disease [6,25]. AVSc is an echocardiographic finding not well understood, which remains a very controversial topic considering it has no clinical symptoms but is linked to increased cardiovascular risk and associated mortality [26,27,28]. Taken together, these data suggest that P2RY2 selective activation should be further investigated as a pharmacological target for the prevention of CAVS progression, acting on both calcification and myofibroblastic activation of VICs. These data could also be of high importance for the future development of tissue engineering. Indeed, 2ThioUTP administration could be proposed in multiple applications, such as maintaining an inactive VIC phenotype and reducing aortic valve fibro-calcification while seeding in a decellularized scaffold for the generation of new tissue-engineered aortic valves.

### Study Limitations

Further studies are needed to better investigate the molecular pathway activated by 2ThioUTP and to prove its antifibrotic effects in vivo. In this regard, the multi-omics approaches implemented in the last few years to study the CAVS progression [11,17,29] could unveil if P2RY2 plays a crucial role in maintaining the aortic valve ECM homeostasis. We know that sex influences the development and progression of CAVS, in terms of both calcium and fibrotic tissue deposition, and the majority of patients enrolled for this study were men [30]. However, men show a higher amount of extracellular calcium; hence, the reduction in calcium exerted by 2ThioUTP reveals its capabilities.

## 5. Conclusions

In conclusion, our findings reveal the potential therapeutic effects of 2ThioUTP in CAVS patients, even at the early stage of the pathology. Its ability to prevent calcium deposition could be of interest to fill the absence of pharmacological treatments for patients with AVSc and avoid the CAVS onset, thus reducing the need for more invasive approaches such as surgery or transcatheter aortic valve replacement. Moreover, these data could also be of high importance for the future development of tissue engineering, considering its capacity to maintain the inactive form of CAVS VICs.

## Figures and Tables

**Table 1:** Patients’ characteristics.

Variables	Patients (*n* = 33)	AVSc (*n* = 13)	AS (*n* = 20)	*p*-Value
Male sex, *n* (%)	25 (75.5)	10 (76.9)	15 (75.0)	>0.99
Age (years), mean ± SD	68.7 ± 9.4	64.1 ± 11.7	71.2 ± 6.8	0.06
Height (m), mean ± SD	1.7 ± 0.1	1.7 ± 0.1	1.7 ± 0.1	0.45
Weight (kg), mean ± SD	79.5 ± 14.2	77.3 ± 15.0	81.0 ± 13.8	0.48
BMI (kg/m^2^), mean ± SD	27.3 ± 4.1	26.0 ± 3.9	28.2 ± 4.0	0.13
Diabetes, *n* (%)	2 (6.1)	0 (0)	2 (10)	>0.99
Hypertension, *n* (%)	23 (69.7)	9 (69.2)	14 (70.0)	>0.99
Dyslipidemia, *n* (%)	19 (57.6)	8 (61.5)	11 (55.0)	0.74
Smokers, *n* (%)	5 (15.1)	2 (15.4)	3 (15.0)	>0.99
CAD, *n* (%)	11 (33.3)	5 (38.5)	6 (30.0)	0.71
PAD, *n* (%)	6 (18.2)	1 (7.7)	5 (25.0)	0.36
TAV morphology, *n* (%)	27 (81.8)	11 (84.6)	16 (80.0)	>0.99
LVEF (%), mean ± SD	59.5 ± 9.3	55.8 ± 9.5	61.9 ± 8.7	0.07
AV Velocity max (m/s), mean ± SD	3.5 ± 1.2	2.2 ± 0.50	4.2 ± 0.8	**<0.0001**
AV Gradient max (mmHg), mean ± SD	53.1 ± 33.1	19.8 ± 9.0	73.1 ± 24.9	**<0.0001**
AV Gradient med (mmHg), mean ± SD	35.3 ± 18.5	12.0 ± 5.0	42.3 ± 14.9	**<0.0001**
Area (cm^2^), mean ± SD	1.1 ± 0.5	2.0 ± 0.2	0.9 ± 0.2	**<0.0001**

BMI: body mass index, CAD: coronary artery disease, PAD: peripheral artery disease, AS: aortic stenosis, AVSc: aortic valve sclerosis; TAV: tricuspid aortic valve, LVEF: left-ventricular ejection fraction, AV: aortic valve.

## Data Availability

Data generated for this study are available upon reasonable request to the corresponding author P.P. (paolo.poggio@ccfm.it).
